# Enterocutaneous fistula as a complication of laparoscopic cholecystectomy

**DOI:** 10.4103/0972-9941.41951

**Published:** 2008

**Authors:** Jeremy Huddy, Sharan S Wadhwani, Yuen Soon

**Affiliations:** Minimal Access Therapy Training Unit, Department of Upper GI and Laparoscopic Surgery, Royal Surrey County Hospital, Guildford, Surrey GU2 7XX, United Kingdom

**Keywords:** Bowel perforation, complications, laparoscopic cholecystectomy

## Abstract

Laparoscopic cholecystectomy is the gold standard method for treating gallstone related disease. Despite its widespread and well established application, clear consensus is not arrived at regarding the comparative risks and benefits of acute versus interval cholecystectomy. The complications of this technique are well known, with respect to both the operative intervention and the technique used. This case describes a case of cholecystitis in a 76-year-old man, who underwent acute laparoscopic cholecystectomy for cholecystitis refractory to antibiotic therapy. Postoperative complications included subhepatic collections bilaterally, eventually leading to the formation of an enterocutaneous fistula to the left chest wall - a previously undocumented phenomenon. The protracted course of the disease is discussed, with reference to investigations performed and the eventual successful outcome.

## INTRODUCTION

Laparoscopic cholecystectomy is considered as a gold standard treatment for gallstone related disease. Conflicting opinions exist concerning the timing of operative intervention in relation to acute cholecystitis. Advantages of early intervention include symptomatic relief and prevention of further painful episodes. However, this is hampered by the significant increase in the conversion rate to open cholecystectomy.[1]

Common complications of laparoscopic cholecystectomy may be related to the approach or the procedure itself. Laparoscopic complications occur in 0.25% of cases and include vascular injuries, bowel injuries and haemorrhage.[1] Common bile duct injury (0.23%), bile leakage and general surgical complications are well-recognised sequelae of the procedure itself.[1] Less commonly associated complications include fistula formation, endoclip migration, cholangitis, bowel injury and intra-abdominal abscess formation.[1] It is suggested that the majority of these complications may be attributable to aetiologies involving ongoing postoperative bile leakage; interestingly Hobbs *et al*, found intraoperative cholangiogram to be associated with a 30% lower risk of all intraoperative complications.

The current case describes a previously undocumented complication of enterocutaneous fistula to the left chest wall following laparoscopic cholecystectomy. The protracted disease process spanned three years, and involves various contributing pathologies.

## CASE REPORT

An otherwise healthy 76-year-old man was admitted as an emergency to the hospital with right hypochondral pain in keeping with cholecystitis. There was no evidence of jaundice or abnormal liver function tests and ultrasound investigation of the abdomen confirmed the presence of gallstones, with a thickened gallbladder wall but normal common bile duct diameter. Acute management involved analgesia, intravenous fluids and antibiotics (erythromycin, given his allergy to penicillin). The patient was submitted for a laparoscpic cholecystectomy when he failed to settle on conservative therapy. It was a technically difficult operation due to the degree of inflammation. The gallbladder was perforated and a subtotal cholecystectomy was performed leaving Hartmans Pouch. Stone clearance was not attempted, due to the absence of deranged liver function tests and the degree of inflammation/infection. The operative field and sub-phrenic spaces were thoroughly irrigated with 5 litres of saline and two wide bore drains were left. 

The patient was discharged on the seventh postoperative day. He was readmitted on day 11 with general malaise, right upper quadrant abdominal pain, shortness of breath and oliguria. A CT scan revealed bilateral pleural effusions and a right subphrenic collection which, when radiologically drained, revealed frank pus. Further CT scan on the 25^th^day demonstrated two further collections in the left sub-phrenic and epigastric regions which were radiologically drained. The patient was eventually discharged on the 53^rd^day.

Over the following two years, this gentleman had multiple admissions for a discharging sinus in his left chest wall. He underwent repeated radiological drainage with antibiotic cover. Cardiothoracic referral was made and three attempts were made to explore the sinus surgically. These explorations involved extensive excision of sinus tract and segmentectomy of the 10^th^rib. Laparoscopy and investigative laparotomy were not implemented given the pathology was identified using conventional radiological techniques. 

**Figure 1:**
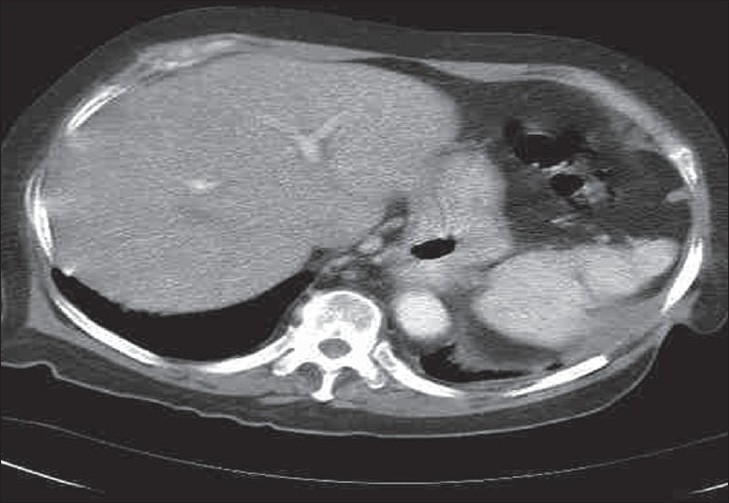
A computed tomograph in the transverse plane, demonstrating a collection of fluid lateral to the spleen

**Figure 2:**
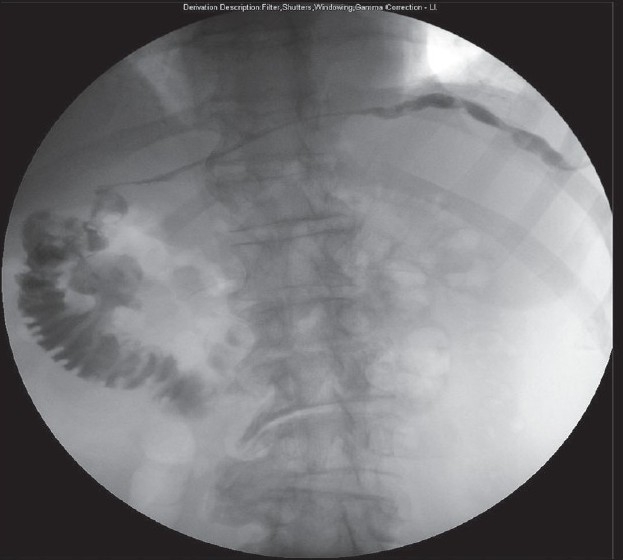
A radiographic sinugram, clearly demonstrating the fistulous tract communicating with the second part of the duodenum

Three years post-surgery, the patient was admitted with a similar presentation. CT revealed the persistence of fluid lateral to the spleen [Figure 1]. A sinugram identified the sinus tract extending from the left chest wall, to the second part of the duodenum [Figure 2]. At laparotomy the fistula was dissected from the first part of the duodenum to the left upper quadrant requiring an awkward dissection of the stomach from the left lobe of the liver to reveal the fistulous track. Finally, the fistula remnant through the diaphragm and lateral chest wall was irrigated and sclerosed with Hydrogen Peroxide. After a rapid, uneventful recovery, the patient is currently well with no further symptoms. 

## DISCUSSION

Damage to the bowel following laparoscopic cholecystectomy is rare.[1] Injury can be caused directly by insertion of laparoscopic trochar or veress needles.[1] However, perforation is more commonly brought about by electrocautery with diathermy.[2] Damage through cautery generally has a delayed presentation as diathermy-induced coagulative necrosis manifests late.[2] The duodenum is at risk of damage from diathermy by inappropriate use of cautery when dissecting Calot's triangle.[2] The duodenum is also at risk of conduction burns as the energy dissipates along the bile duct.

Typical symptoms of bowel perforation are abdominal pain, peritonitis and ileus associated with sepsis.[3] Bishoff *et al*, suggest that the majority of patients do not present in this typical fashion. Indeed it is suggested that pain surrounding the trocar site closest to the perforation, low-grade pyrexia, diarrhoea and abdominal distension are much more common presentations.[3] Leukopenia appears more common than leukocytosis associated with low-grade pyrexia.[3]

Enterocutaneous fistulae arsing from the duodenum are rare.[4] However, they are associated with a greater morbidity and mortality as the surrounding tissues are exposed to high amounts of enzyme rich secretions.[4] Mortality rates in such patients are high, in the order of 32-33%.[4] The management of such fistulae should target the symptoms of sepsis, malnutrition, skin excoriation and fluid and electrolyte balance.

In conclusion, a high index of suspicion and vigilant physical examination is imperative to the early recognition of bowel damage. Laboratory and radiographic investigations have been found to be non-specific.[5] It is suggested that any suspicion of intraabdominal sepsis following laparoscopic cholecystectomy should warrant early repeat exploratory laparoscopy to ensure diagnosis, abdominal toilet and hopefully repair.
